# Evaluation of force pain thresholds to ensure collision safety in worker-robot collaborative operations

**DOI:** 10.3389/frobt.2024.1374999

**Published:** 2024-04-08

**Authors:** D. Han, M. Y. Park, J. Choi, H. Shin, R. Behrens, S. Rhim

**Affiliations:** ^1^ Department of Mechanical Engineering, Kyung Hee University, Yongin-si, Republic of Korea; ^2^ Robotic Systems, Fraunhofer IFF, Magdeburg, Germany; ^3^ Department of Industry-Academic Cooperation Foundation, Kyung Hee University, Yongin-si, Republic of Korea; ^4^ Safetics, Seoul, Republic of Korea

**Keywords:** safety, physical human-robot interaction, collision safety, biomechanical limits, pain threshold, impact

## Abstract

With the growing demand for robots in the industrial field, robot-related technologies with various functions have been introduced. One notable development is the implementation of robots that operate in collaboration with human workers to share tasks, without the need of any physical barriers such as safety fences. The realization of such collaborative operations in practice necessitates the assurance of safety if humans and robots collide. Thus, it is important to establish criteria for such collision scenarios to ensure robot safety and prevent injuries. Collision safety must be ensured in both pinching (quasi-static contact) and impact (transient contact) situations. To this end, we measured the force pain thresholds associated with impacts and evaluated the biomechanical limitations. This measurements were obtained through clinical trials involving physical collisions between human subjects and a device designed for generating impacts, and the force pain thresholds associated with transient collisions between humans and robots were analyzed. Specifically, the force pain threshold was measured at two different locations on the bodies of 37 adults aged 19–32 years, using two impactors with different shapes. The force pain threshold was compared with the results of other relevant studies. The results can help identify biomechanical limitations in a precise and reliable manner to ensure the safety of robots in collaborative applications.

## 1 Introduction

With advancements in robot technologies, robots have been increasingly applied in various fields (Billard and Kragic, 2019). Especially, robots are being widely used in the industrial sector (Villani et al., 2018), in close proximity with human workers (Ferrández et al., 2013). A notable development is the concept of collaborative operations, which can serve as a bridge between simple repetitive tasks performed by robots and highly skilled manual tasks performed by human workers (Krüger et al., 2009; Wang et al., 2019; Behrens et al., 2022). However, such operations involve risks of injury to humans owing to frequent contact between the robots and the human body (Bicchi and Tonietti, 2004; Haddadin et al., 2009). Therefore, many researchers have focused on human collision safety (Haddadin et al., 2007; Oberer and Schraft, 2007; Haddadin et al., 2010; Su et al., 2018; Su et al., 2019; Behrens and Elkmann, 2021). As a preliminary measure to address the safety concerns, the standard ISO/TS 15066 was established to provide safety guidelines for the collaborative operation of industrial robot. To clarify safety criteria for collisions, the biomechanical limitations have been measured (Yamada et al., 1997; Melia et al., 2015; Park et al., 2019; Behrens et al., 2022; Han et al., 2022). For example, in addition with clinical trials, collision experiments with porcine skin tissue with properties similar to the human skin were conducted to identify the propensity for injury based on mechanical parameters (Mao et al., 2017; Han et al., 2018; SUGIURA et al., 2022). Moreover, through clinical analysis of the collided porcine soft tissue, the collision conditions (e.g., effective mass of the robot, impact velocity, and geometry) that could lead to injury were quantified and applied to robot safety control (Haddadin et al., 2012). Similarly, clinical analysis of subcutaneous tissue injured by collisions was performed to determine the energy density (energy transferred per unit area) that could lead to internal bleeding (Povse et al., 2010; Fujikawa et al., 2017), given that the energy density is considered a cause of contusion in forensic sciences (Desoulin and Anderson, 2011).

Ensuring the safety of robots and humans in collision scenarios is essential to promote the implementation of collaborative applications in various fields (Kulić and Croft, 2007). In the case of physical contact between a robot and human leading to injury, two situations can be identified: impact (transient contact) and pinching (quasi-static contact) (Ferraguti et al., 2019; Behrens et al., 2022), and thus, the collision safety must be ensured in both situations. To evaluate the safety, it is necessary to assess the level of injury. As a representative technique for such assessments, virtual contact sensors were used to evaluate the contact force and pressure before implementation of collaborative operations (Shin et al., 2019). Moreover, as mentioned previously, clinical trials were conducted to measure the pain initiation thresholds in pinching situations. For impact situations, the biomechanical limits have been specified in ISO/TS 15066. These criteria were derived from extensive literature surveys. Subsequent studies were focused on evaluating the biomechanical limits by simulating impact with different parts of the human body (Behrens and Elkmann, 2014; Behrens et al., 2022).

In this study, a clinical trial for evaluating mechanical pain caused by impacts was conducted. The results of the clinical trial were compared with similar data from existing research. An apparatus was designed to conduct the initial part of the study. This apparatus could implement impact situations to measure the mechanical pain initiation thresholds resulting from a collision between humans and robots. Specifically, the impact was applied using a test apparatus with a pendulum-type impactor with a speed of up to 3.3 m/s. The maximum permissible contact force from the impact was derived to quantify the associated pain (Williamson and Hoggart, 2005), and partial biomechanical limitations that can be used to verify the safety of industrial robots operating in collaborative applications were statistically analyzed. The outcomes can provide guidance for establishing safety standards for the collaborative operation of industrial robots in the future.

## 2 Materials and methods

As part of efforts to ensure safety from industrial robots in collaborative workspaces, ISO/TS 15066 presents four methodologies for collaborative operations. Among these methods, the power and force limiting (PFL) mode can safeguard robots from injury caused by collision accidents. The mode ensures that the robots do not exceed the biomechanical limitations, by limiting the operating speed or modifying the robot posture. To this end, ISO/TS 15066 presents the biomechanical limitations based on the pain and injury onset for the application of the PFL mode. In this context, it is necessary to maintain the appropriate biomechanical parameters, such as the pressure and force pain thresholds. The clinical trial in this study was designed to acquire further information related to the force pain thresholds in impact situations.

The Institutional Review Board (IRB) of Kyung Hee University Hospital approved all processes. The protocols and setup were designed with reference to other relevant studies (Yamada et al., 1997; Behrens and Elkmann, 2014). The entire procedure of the clinical trial, including the research protocol and the case report form, was submitted to the IRB for approval (IRB File No. 2018-11-023). The new application received qualified permission with review comments related to subject selection exclusion criteria, recruitment advertising, and insurance coverage. Subsequently, corrective and preventive action plans were submitted. After reviewing the amended protocol as instructed by the IRB review, the clinical trial of this study was approved (IRB File No. 2018-11-023-003). In the recruitment process, the potential subjects were informed of the clinical trial protocol and insured against possible injuries. The treatment for all subjects was adequately documented. After the clinical trial ended, the clinical trial report was submitted and approved (IRB File No. KHUH 2018-11-023-019).

### 2.1 Pain threshold assessment apparatus

**FIGURE 1:**
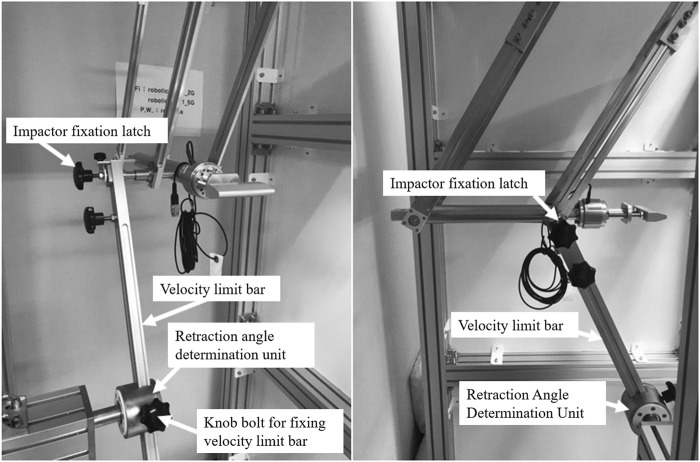
Force pain threshold assessment apparatus. The pendulum arm structure consists of 4 bars that allow the impactor to be parallelly aligned with the ground.

The experimental device for implementing dynamic collision situations contains a pendulum arm. The arm consists of 4 bars connected to the impactor body by bearings, allowing the impactor body to remain level with the floor. Measurement devices, including a six-axis force/torque sensor (ATI Industrial Automation, NC, United States, Mini58; range ±48,000 N) and laser displacement sensor (KEYENCE, Japan, LK-G150; range ±40 mm), are attached to measure the contact force and velocity during the collision, respectively. The data generated by this device are stored on a computer through a data acquisition system (NI, TX, United States SCB-68A). In the experimental apparatus designed to measure contact forces and displacements, the integration of a load cell and a laser sensor into a cohesive measurement framework was paramount for capturing accurate and concurrent data. To achieve this, both sensors were interfaced with a unified data acquisition system, meticulously configured to record data at a both same sampling frequency of 10 kHz. Figure 1 shows the impact assessment system designed for the clinical trial.

**FIGURE 2:**
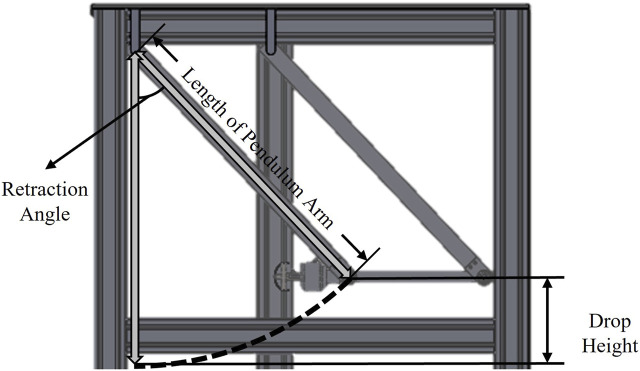
Schematic of pendulum arm type impactor. The impactor collides with the body locations for pain assessment when the pendulum arm is perpendicular to the ground.

The impactor is retracted through the calculated angle to generate a precondition for applying the impact on the subject at a velocity that satisfies the required velocity. The position of the retracted impactor is secured by placing the fixation latch. After retracting the impactor, the body location for pain assessment is positioned at the lowest position along the trajectory of the impactor. When the fixation latch is removed, impact is generated by the motion of the pendulum along a circular trajectory, under the influence of its weight and gravity. The impactor collides with the human subjects when it reaches the lowest position, i.e., when the pendulum arm is perpendicular to the ground. The structure of the test apparatus is shown in Figure 2. The potential energy of the impactor at the initial position, defined by the fixation latch, can be estimated based on the drop height. The retraction angle necessary to achieve the target impact velocity can be calculated by assuming that the potential energy is fully converted to kinetic energy when the impactor reaches the lowest point. Through these fundamental principles, the impact velocities are determined and adjusted by varying the retraction angle.

Although the retraction angle is determined theoretically, friction in the bearings connected to the pendulum arm may lead to differences between the theoretically predicted impactor velocity and the actual value. However, because the objective is to identify the mechanical input that leads to pain by incrementally increasing the rate of collisions, the trend of increase in the impact velocity is considered to be more important than the application of the exact velocity. The incremental increase in the impact velocity trend is confirmed through monitoring during the clinical trial.

**FIGURE 3:**
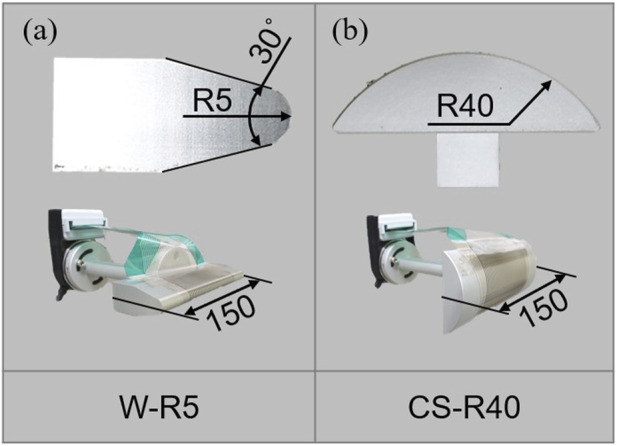
Side view of the contact surfaces used in clinical trials. **(A)** Wedge shape with an edge radius of 5 mm (W-R5) **(B)** Cylinder with a radius of 40 mm (CS-R40).

Two types of impactors with various shapes of the contact surface are considered: wedge-shaped (W-R5), and cylindrical (CS-R40) (Figure 3). Both shapes are selected empirically to mimic the external surfaces of commonly encountered off-the-shelf industrial robots that can operate without safety fences. All shapes are composed of the 6061-aluminum alloy and designed to have the same weight. CS-R40 have a radius of 40 mm, and W-R5 has an edge radius of 5 mm. Figure 3 shows the impactor shapes, aligned perpendicular to the body locations. A film-type pressure sensor, which was affixed to the surface of the collision geometry, was employed in an attempt to measure the pressure dynamics during impact. However, this approach encountered several notable challenges. Firstly, the characteristics of the collision interface posed substantial difficulties in ensuring complete and uniform coverage by the sensor. This was crucial, as incomplete coverage could lead to inaccurate or partial data that did not capture the full spectrum of pressure variations throughout the contact area. Secondly, the inherently brief duration of the contact events further compounded the issue. Although the sensor response is rapid, it was likely not sufficiently swift to accurately capture the transient pressure peaks characteristic of such high-speed impacts. As a result of these limitations, the data obtained from the film-type pressure sensor did not yield significant or reliable values. After consideration and thorough analysis, it was concluded that including these data would not contribute meaningfully to the understanding of collision dynamics, and these measurements were excluded from the analysis presented in this study.

### 2.2 Subjects

Subjects for this clinical trial were recruited through online and offline announcements and advertisements. Table 1 summarizes the information of the selected participants. The age range was 19–32 years. Due to a limited number of female applicants, only male subjects were recruited. Individuals with a medical history, drug abnormalities, (acute) illness, medication intake, severe psychological or mental problems, allergic contact dermatitis related to metals, pain or sensory anomalies, and acute infections were excluded. Similarly, individuals with chronic diseases such as diabetes, hypertension, stroke, arrhythmia, ischemic heart disease, malignant tumor, allergic diseases, nervous system disorders, musculoskeletal diseases, chronic obstructive pulmonary diseases, and asthma were excluded. To ensure the fairness of the results, students from the Department of Mechanical Engineering and the College of Medicine of Kyung Hee University were not allowed to participate in the clinical trial. Before the clinical trial, the methods and objective were explained to the subjects to ensure the accuracy of the results. All subjects were adults over the age of 18 and provided written informed consent for participation. Moreover, the subjects were educated about the pain scale in advance to help them understand the pain onset. The tools used to this end were the visual analog scale and abbreviated injury scale (Greenspan et al., 1985; Williamson and Hoggart, 2005).

**TABLE 1:** Characteristics of the subjects.

Characteristics	Description
Number of participants (n)	37
Age	19–32 years (average 25 years)
Sex	Male
Weight	47–100 kg (70.1 ± 9.9 kg)
Height	160–182 cm (173.6 ± 4.7 cm)
BMI	18.6–30.5 (23.2 ± 2.7)

### 2.3 Procedure of the clinical trial

**FIGURE 4:**
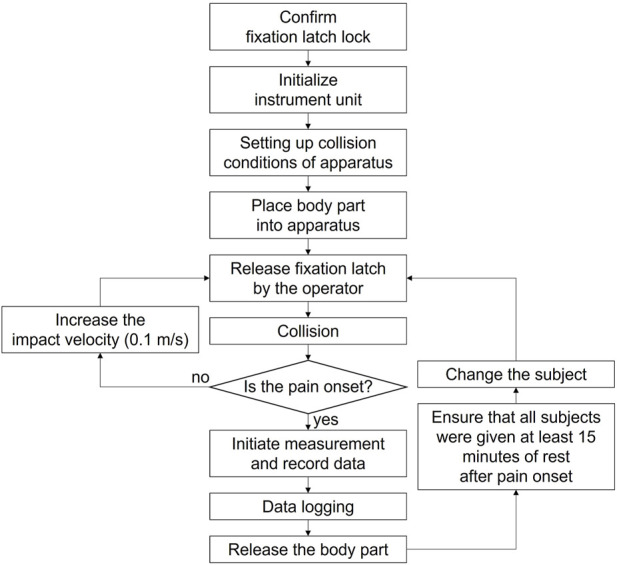
Process flow of the clinical trial.

The force pain thresholds were measured through the process illustrated in Figure 4. The impactor was constrained by the fixation latch for safety, except when it generated the pendulum motion to apply impact. Theoretically, the maximum impact velocity was 3.3 m/s, but a value of 2 m/s was set for the tests. The maximum impact velocity of 2 m/s was set considering the results of a previous study on skin injury based on minipig skin tissue (Han et al., 2018). In the first trial, the subjects filled out questionnaires regarding their body size, vital signs, current medical conditions and medications, drug abnormalities, allergies to metals, sleep patterns, and mood conditions. The experimenters reviewed the questionnaires to identify any symptoms included in the exception list. Subsequently, the pain resulting from impact collisions with each body location was measured through the pain threshold assessment apparatus. In the second trial, impact was implemented only when there was no residual sensation of pain or compression remaining on the skin of the subjects.

**FIGURE 5:**
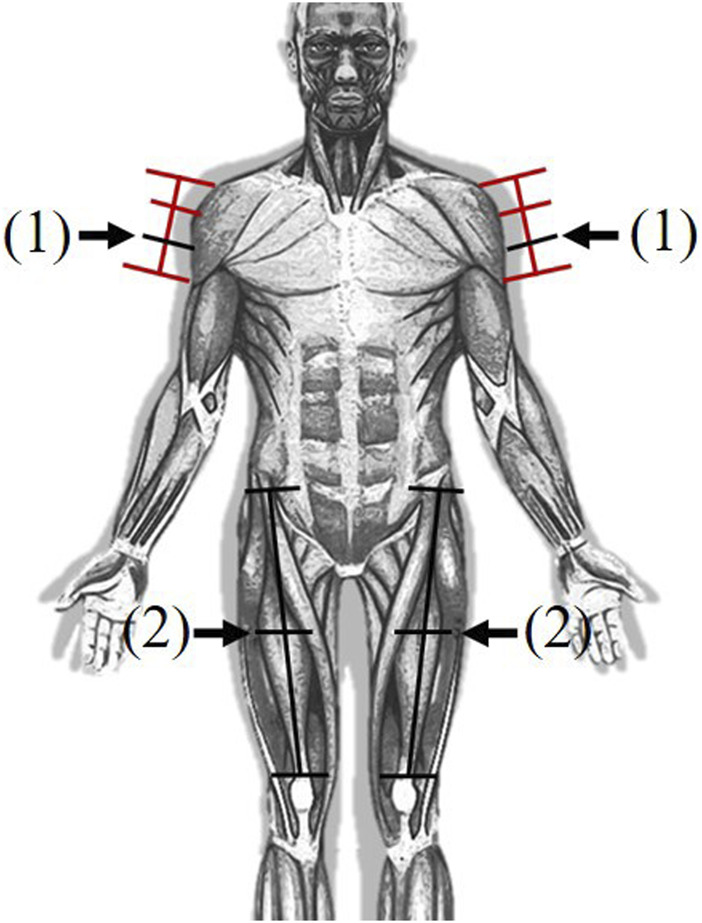
Body locations for pain measurement. (1) Deltoid (2) Thigh.

The procedure for the first trial involved the following steps. As a first step, pain measurements associated with impact with W-R5 or CS-R40 were conducted on two body locations: the deltoid and thigh. After the impact and force measurement process, subjects were provided with sufficient break time. In a single trial, the deltoid and thigh were subjected to impact with a single contact surface. To obtain conservative values for the pain threshold, measurements were performed on the nondominant deltoid and thigh (Kosek et al., 1993; Özcan et al., 2004). The second trial followed the same steps, albeit with the impact condition not considered in the first trial. Consequently, in the case of impact on the deltoid and thigh, the measurements were obtained twice, each time using an impactor with a different shape. The body locations are shown indicated in Figure 5.

The contact force was measured until the pain threshold was reached, i.e., when the subject experienced mechanical pain on the skin. The impact velocity was increased from 0.8 m/s to the maximum value in intervals of 0.1 m/s. The subjects were repeatedly subjected to impact on the same body location, with increasing impact velocity. This collisions were terminated when the subjects reported pain or a residual sensation lasting more than 15 min. The existing studies have indicated that the time interval between consecutive impacts under increasing impact velocity is not strongly correlated with the pain threshold (Isselée et al., 1997; Chesterton et al., 2003; Behrens et al., 2022). For each collision, the contact force and impact velocity were monitored and recorded using a computer connected to the sensors in the apparatus.

### 2.4 Body locations

**FIGURE 6:**
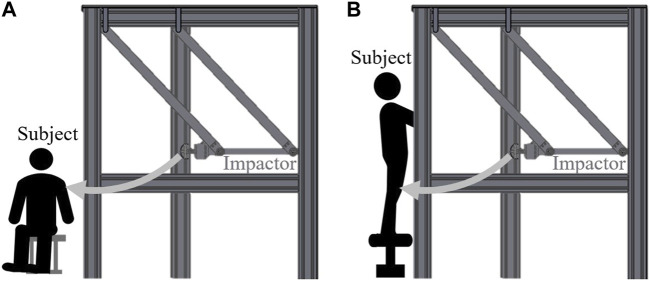
Schematic of impact situations for selected body locations. **(A)** Deltoid **(B)** Thigh.

We selected two regions of the body for clinical evaluation of pain, based on the biomechanical limits presented in ISO/TS 15066. Due to budget and time constraints, tests could not be conducted for all the body locations. Therefore, the body locations at which pain is easily felt or those associated with additional risk were identified and excluded. Most areas adjacent to the cervical spine were noted to be highly pain sensitive, and injuries to terminal extremities could interfere with daily life. Additionally, body locations that could be injured immediately after impact were excluded. For example, the hand and pelvis with protruding bones susceptible to fractures were excluded. Considering these aspects, the deltoid and thigh were selected appropriate locations to measure the pain threshold under impact (Figure 5). The specific measurement locations over these parts are summarized in Table 2. When the deltoid muscle, situated on the upper arm, is subjected to an impact, the impactor is positioned so that it is perpendicular to the sagittal plane of the body. Conversely, for impacts involving the thigh, the impactor’s alignment is adjusted to be perpendicular to the frontal plane. This setup is guided by the specifications outlined in ISO/TS 15066. The contact surface between the impactor and pain measurement area were always parallelly aligned. Moreover, the subject’s body parts were not fastened during the collusion to simulate real-world scenarios in the industrial field. The impact situations for each body location are shown in Figure 6. The postures of the subjects were set based on the selected body locations and apparatus structure.

**TABLE 2:** Body locations for pain assessment.

Body location	Description
Deltoid	Lower 1/3 point of the deltoid area in the upper arm
Thigh	Midpoint of the line connecting the upper part of the patella in the anterior superior iliac spine

For the trial of deltoid impacts, subjects were seated comfortably in a chair, adopting a posture that allowed the arm to be in a relaxed state. This positioning facilitated a natural resting position for the arm, ensuring that the muscle tone and orientation of the deltoid were representative of typical everyday scenarios. In contrast, for the assessment of impacts on the thighs, a different approach was taken. The subjects were placed in a standing posture, which is a more representative posture for the impacts that could occur to the lower body during activities. To maintain balance and ensure safety, subjects were instructed to hold onto a support with their arms. This precaution was necessary to prevent the subjects from falling as a result of the impact, thereby minimizing the risk of injury during the experiment. The standing position, coupled with the provision for stability, allowed for a natural alignment and loading of the thigh muscles, akin to the conditions under which thigh impacts typically occur in real-life situations. Because the primary focus was on measuring the biomechanical thresholds to mechanical forces under impact conditions, the post-collision behavior of body parts was not measured.

### 2.5 Statistical analysis

The data collected during the clinical trials were analyzed using statistical methods. Specifically, quantile regression, probability distribution fitting, and calculation of the cumulative distribution function (CDF) were realized using R (R Foundation, Austria, ver. 3.4.3) and MATLAB (MathWorks, CA, United States R2022b). The distribution was estimated based on 37 measured pain thresholds to the extract significant third quartiles as biomechanical limits and compared with the distribution of the original results. This process of estimating statistical models allows compensation for the error that sample size can introduce. Moreover, the results were compared with those of other studies.

## 3 Results

In the clinical trials, four impact cases were simulated per subject. Therefore, the maximum contact force and response contact velocity were recorded from 148 trials. A temporal analysis focusing on the duration from the onset of an impact event to the moment when the peak force was registered was also conducted. When utilizing the CS-R40 contact surface against the deltoid muscle, the average time elapsed until the peak force was achieved was recorded at 28.5 milliseconds (msec). The time required for the same impactor to reach the peak force when colliding with the thigh, which was slightly longer, averaged 34.6 msec. Furthermore, when the impacts were administered using the W-R5 contact surface, the average time to reach peak force upon impacting the deltoid muscle was 34.9 msec, whereas, for thigh impacts, this time extended to 40.6 msec. The measured results were statistically analyzed, the skin injuries were visually inspected. The representative values derived from the statistical analyses were used to identify the biomechanical criteria to ensure the safety of robots in collaborative applications.

### 3.1 Skin damage

In the event of pain onset, the skin of the impacted area was photographed immediately after collision. The occurrence of skin injury (erythema) was confirmed by evaluating the redness of the skin in consultation consulting with the dermatologist at Kyung Hee University Medical Center. Erythema was observed in two cases of collision between the thigh and W-R5 contact surface. No severe reaction (e.g., bruises or contusions) were observed in these cases.

### 3.2 Force pain thresholds

The contact forces generated by the impacts were measured using a load cell attached to the experimental apparatus. The data measured from 37 subjects according to the body location and impactor type were acquired. Values for each impact case were obtained once for each subject. The lower contact force initiated pain typically when the contact surface was sharp. Thus, in impact situations, it may be challenging to ensure the collision safety of industrial robots with various shapes using a single biomechanical limit value for each body location.

### 3.3 Descriptive statistics

**FIGURE 7:**
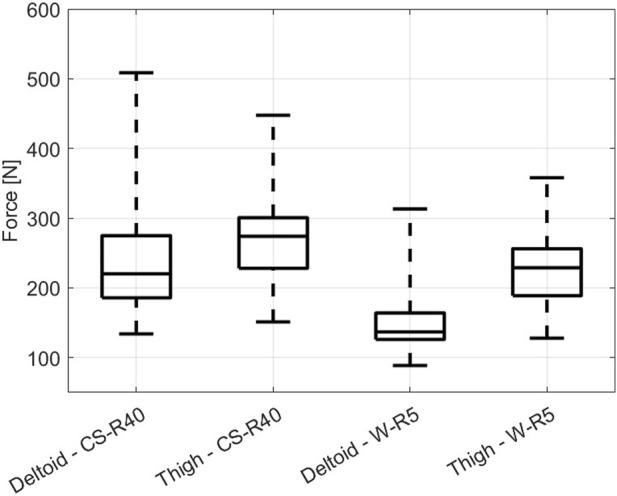
Boxplots showing the force pain thresholds of the contact force measured from 37 subjects.

Biomechanical data obtained from the 37 subjects were subjected to probability distribution fitting and quantile regression for generalization. The impact velocity was gradually increased by 0.5 m/s for each impact condition until pain onset. Because this increase in velocity was not continuous, the exact force pain threshold was expected to lie between contact forces from the final and penultimate test. Midpoint imputation was applied to estimate the unknwon contact force (Sun, 2006; Behrens et al., 2022). The concept of midpoint imputation can be expressed as

$$FPT_{mi}=\left\{\begin{array}{cc} \frac{F_{h}}{2}\; & \text{if }F_{p}=0\\ F_{p}\; & \text{if }F_{h}\to \infty \\ \frac{F_{p}+F_{h}}{2}\; & \text{otherwise} \end{array}\right.$$ (1)

where *FPT*_*mi*_ is the estimated force pain threshold in the case of midpoint imputation; and *F*_*h*_ and *F*_*p*_ are the highest and penultimate contact force values, respectively. *F*_*p*_ = 0 means that the subject felt pain in the first impact trial without any increase in the impact velocity. *F*_*h*_ → *∞* means that pain does not onset despite the impact load reaching the maximum level. The graph in Figure 7 shows the results obtained after updating the data with midpoint imputation described in Eq. 1, and the corresponding descriptive statistics for the box plots are presented in Table 3. The distribution model is derived from the data with midpoint imputation.

**TABLE 3:** Descriptive statistics for the force pain threshold derived through data subjected to midpoint imputation.

Impactor **shape**	Body	Force pain threshold [N]				
	**Location**	Min	Q1	Q2	Q3	Max
CS-R40	Deltoid	134.1	185.8	220.2	274.9	508.7
	Thigh	151.2	228.1	274.1	300.7	447.7
W-R5	Deltoid	88.7	126.0	136.9	164.0	313.3
	Thigh	128.0	188.8	228.9	256.2	358.0

Upon examination of the data distribution illustrated in Figure 7, a notable observation emerges regarding the distribution patterns of the biomechanical limits of the deltoid and thigh to external impacts. The similarity in their data scatter can be attributed to the comparative thickness of these muscle groups, which inherently influences their capacity to absorb and dissipate impact forces. In particular, the distribution reveals that despite these similarities, the deltoid muscle exhibits a consistently higher force pain threshold compared to the thigh muscle. This distinction suggests that the deltoid possesses a higher resistance to pain-inducing forces under the conditions tested. The further insights derived from Figure 7 relate to the influence of contact geometry on the initiation of pain. The force pain thresholds are generally lower when the contact surface is relatively sharper, as exemplified by the comparison between the W-R5 and CS-R40 impactors. The W-R5, being sharper, tends to concentrate the applied force on a smaller area, thus increasing the localized pressure and reducing the force required to reach the pain threshold. On the contrary, the CS-R40, with a less acute contact geometry, distributes the force over a larger area, necessitating a higher force to achieve similar levels of pain sensation.

### 3.4 Distribution model

The relevant distribution model for the force pain threshold is considered to estimate the quantiles related to the biomechanical limitations. The inverse of the CDF is used to predict the distribution quantiles. Specifically, CDF *F* is defined as

$$F\left(y_{f}\right)=q$$ (2)

where *y*_*f*_ is the contact force value, which is the variable; and *q* is the quantile 0 < *q* < 1. The value of the contact force as a variable to estimate *q* can be expressed as the inversion of Eq. 2

$$y_{f}=F^{-1}\left(q\right)\;.$$ (3)

**FIGURE 8:**
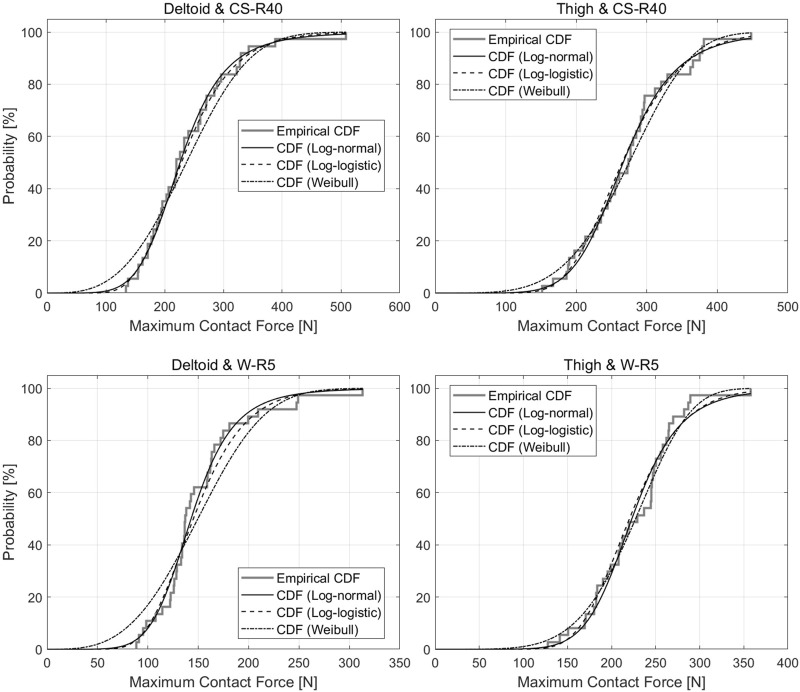
Cumulative Distribution Function and Empirical Distribution Function for the results of each impact case in the clinical trial.

The log-normal, log-logistic, and Weibull distribution models are the most commonly used distribution models for the CDF in biomechanical data analysis (Kent and Funk, 2004; Behrens et al., 2022). CDFs based on these distribution models are generated, and the Anderson–Darling test is conducted to verify the fitness of the empirical distribution function (EDF) from the measured data. The log-logistic distribution model exhibits the closest fit with the EDF, as shown in Figure 8. Using Eq. 3, the quantiles are calculated from the CDF with the log-logistic distribution (Table 4). The third quantile is relevant to the biomechanical limitation of pain onset (Behrens et al., 2022; Han et al., 2022).

**TABLE 4:** Summary statistics for the cumulative distribution function with the log-logistic distribution model.

Impactor **shape**	Body	Force pain threshold [N]		
	**Location**	Q1	Q2	Q3
CS-R40	Deltoid	188.3	226.2	271.8
	Thigh	228.7	266.6	310.8
W-R5	Deltoid	121.7	142.9	167.8
	Thigh	195.1	223.1	255.0

## 4 Discussion

In this study, a clinical trial was conducted to determine biomechanical limitations to ensure the collaborative application of industrial robots. The key results pertaining to these limitations and their implications are discussed in this section.

### 4.1 Objectives

It is necessary to identify the biomechanical limits to ensure collision safety in scenarios involving human–robot interactions. Although this study is focused on collaborative operation of robots in industrial scenarios, the force threshold for pain onset can be applied to establish safety guidelines for any machine that operates in close proximity to humans.

To this end, biomechanical limits must be established for all human body locations considering various contact surfaces. However, due to time and budget constraints, this study focused only on impact situations involving two body locations and two contact surfaces. Notably, several researchers have attempted to analyze the biomechanical limits for various body locations (Özcan et al., 2004; Melia et al., 2015; Melia et al., 2019; Park et al., 2019; Liew et al., 2021; Behrens et al., 2022; Han et al., 2022). Overall, the literature reports pain thresholds for approximately 29 body locations. However, because these locations constitute small regions of the human body, a novel method that can identify the pain of the entire human body must be established. Furthermore, the results of most of the existing studies pertain to only pinching or clamping situation. Typically, the pain threshold for impact is approximately two times that for pinching or clamping (Yamada et al., 1997; Behrens et al., 2022). Thus, it is necessary to examine the threshold difference between impact and pinching or clamping situation.

Comprehensive specification of biomechanical limitations that are inherently involved in collaborative operation of human workers and robots can help verify the safety of robot operation through collision tests or simulation methods (Robotic Industries Association, 2018; Shin et al., 2019). For example, although the biomechanical limitations specified in ISO/TS 15066 can be used to validate the collision safety of industrial robots in collaborative workspaces, this standard states that the operational speed of the robot must not exceed the specified limits which may cause the robots to be too slow to be effectively used. To resolve this problem, researchers are exploring the connection between the limitations and alternative thresholds above pain onset (e.g., the maximum bearable pain or injury onset) (Han et al., 2022; Behrens et al., 2023). Future studies can be focused on critically analyzing these considerations to establish safety standards.

### 4.2 Indeterminate errors

As described in Section 2.2, the body parts of the human subjects were not secured against movements in the clinical trial. Consequently, the impacted body parts moved backward in an arbitrary manner in each trial. The experimenters attempted to obtain measurements in identical conditions for each body part of all subjects. However, all contact conditions were not completely identical due to variations in the joint stiffness and tissue properties of individual subjects (Rajaei et al., 2023). In addition, the differences in pain perception among subjects could lead to uncertainties in the outcomes, even when they received the same guidance for determining pain onset. Tolerance and sensitivity to mechanical pain vary because of individual characteristics (Foulkes and Wood, 2008; Lacroix-Fralish and Mogi, 2009; Dubin and Patapoutian, 2010).

To ensure ideal impact conditions for the clinical trial, it is necessary to ensure precise alignment between the impactors and body locations. Because the body part was not physically fixed, misalignment could occur between the impactor surfaces and body locations. The subjects were instructed to maintain a natural response to the impact direction when the impact occurred. However, unintentional movements by the subjects to avoid physical stimulation could introduce variations between trials, potentially influencing the generated contact force and subsequent measurements.

Among the possible errors, an additional consideration is the role of inertial forces that may inadvertently contribute to the force measured by the load cell. This phenomenon is attributed to the mass of the components situated between the load cell and the point of impact on the body during the collision event. In configurations when the W-R5 and CS-R40 are integrated into a pendulum-shaped impactor, the mass contributing to inertial forces is quantified as 0.54 and 0.74 kg, respectively. Given that the total mass of the pendulum system approximates 13 kg, the mass responsible for generating inertial forces constitutes approximately 5 percent of the total system mass. When the pendulum is at its moment of impact, where it reaches its lowest point, the tangential acceleration of the pendulum is theoretically negligible, approaching zero. This assumption simplifies the calculation of the centripetal acceleration, which, in conjunction with the length of the pendulum and the velocity of collision, allows for an estimation of the inertial forces at play. Through this methodology, the average inertial force exerted by the W-R5 on the deltoid muscle was found to be 0.66 and 0.86 N when impacting the thigh. Similarly, for the CS-R40, the calculated average inertial forces were 1.58 N for the deltoid and 1.62 N for the thigh. Despite the identification and quantification of these inertial forces, it is pertinent to recognize their relatively minor magnitude, especially when compared to the force pain thresholds known to induce pain onset. Consequently, the decision was made not to adjust the experimental data for these inertial contributions under the premise that their effect on the overall force measurements is minimal.

### 4.3 Data comparison

To investigate the force or pressure pain threshold related to the onset of pain in impact situations, several studies have been conducted with objectives similar to those of this study (Yamada et al., 1997; Behrens et al., 2022). ISO/TS 15066 provides the pain and force thresholds as biomechanical limitation based on the relevant literature related to pain and injury onset (Deutsche Gesetzliche Unfallversicherung, 2011; Muttray et al., 2014; Melia et al., 2015). Notably, the force pain threshold for impact scenarios may have blind spots due to impractical assumptions. Analyzing and comparing the study cases can potentially help clarify the force pain thresholds.

**FIGURE 9:**
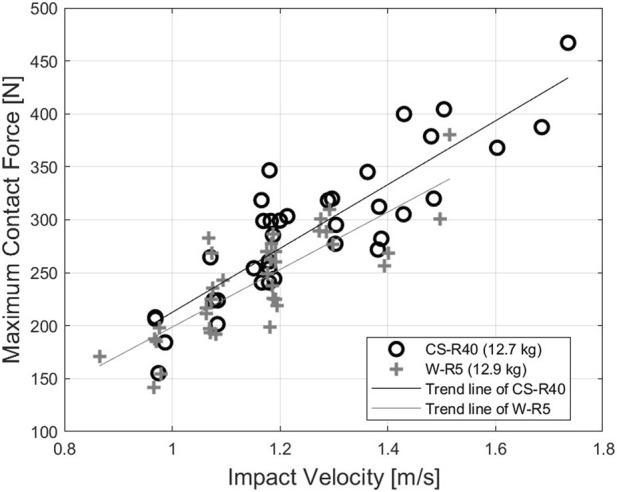
Relation between the impact velocity and contact force for impact at the thigh.

Figure 9 shows the relationship between the impact velocity and maximum contact force based on thigh measurements for different contact surfaces. The overall force values of the impact cases with W-R5 are lower than those with CS-R40, as discussed in Section 3.2. Furthermore, the gradient of the trendlines for the two contact surfaces is similar. This tendency can also be identified in the results obtained for the deltoid.

Behrens et al. (2022) proposed the force pain threshold from a similar experimental setupand attained the same results as those shown in Figure 9. However, the gradient and maximum contact forces values reported by Behrens et al. (2022) are lower than those of this study. This difference can be explained by the different contact conditions between the two studies. In the study of Behrens et al. (2022), the body parts of subjects were physically fixed using straps and vacuum cushions, and the contact surfaces were different than those considered herein. In the pendulum-type impactor, specifically when equipped with the CS-R40 and W-R5 contact bodies, their masses are both approximately 13 kg. Furthermore, this measurement was obtained while subjects were in a standing position, ensuring that the posture and muscle tension reflected a natural stance likely to be assumed during real-world impact events. The data points, represented by black circles and gray crosses in Figure 9 for the cases of CS-R40 and W-R5 impacting to thigh, respectively, were meticulously measured from each subject within our study cohort.

Similarly, the suggested biomechanical values from each study can be compared. In ISO/TS 15066, the force pain thresholds related to injury onset (second column in Table 5) are presented as biomechanical limits. The limit values for impact (transient contact) are calculated as two times the limit values for pinching (quasi-static contact) reported in the literature. Behrens et al. (2022) provide another set of biomechanical limits (fourth column in Table 5) as the 75th percentile of the generated CDF. The result from this study is shown in the third column in Table 5. In the context of defining safety standards and thresholds for human-robot interaction, the 75th percentile, or third quartile, emerges as a pivotal statistical measure. This percentile has been systematically integrated into the biomechanical limits outlined in ISO/TS 15066, which serves as a guideline for the safety of collaborative operation of robot systems. The adoption of the 75th percentile as a benchmark in this standard is informed by empirical research, including studies that have identified this percentile as a critical threshold for the onset of pain in humans upon impact or exertion. The rationale behind selecting the 75th percentile as a biomechanical limit is further supported by the empirical evidence presented in Section 3.1, which documents that only a minimal number of mild injuries were recorded when this threshold was adhered to.

**TABLE 5:** Comparison of biomechanical limit values (75th percentile) for impact with those reported in other studies.

Body **location**	Biomechanical limits [N]		
	ISO/TS 15066	Han et al. (2018)	Behrens et al. (2022)
Deltoid	300	272 (CS-R40)	115
		168 (W-R5)	
Thigh	440	311 (CS-R40)	204
		255 (W-R5)	

As mentioned previously, the contact conditions and premise of this study and that of Behrens et al. (2022) are somewhat different. Among these limits, those specified by the standard, which focuses on the injury onset, are the highest. The second-highest values pertain to this study in which the body parts were not immobilized, and the impact was administered over a large contact area. The lowest limit values are derived from the contact condition in which the body parts were physically fixed, and the impact was delivered through a relatively small contact area, specifically, a 30 mm diameter circle. Although the conditions in which the criteria were derived are slightly different, they were generated to establish the criteria for collision safety in robots. If considering only contact force, pain initiation due to contact force should theoretically commence at a consistent force level across different contact conditions. However, the results of the distinction between similar studies suggest that the principle of pressure also has a significant effect on the mechanism of response to pain. Because the concept of pressure is defined as force per unit area, when subjected to a given force, a smaller contact area will result in a higher pressure than the same force applied over a larger area. This phenomenon assumably explains the observed gap in force pain thresholds. The gender distributions of the subjects and impactor material also likely influenced the results of each study. Considering these aspects, it is essential to conduct research considering both contact force and pressure to establish consistent and precise safety criteria.

## 5 Conclusion

This study was aimed at examining the force pain threshold for impact situations through clinical trials. The force pain threshold can be used as a biomechanical limit when the robot is operating under collaborative operation with power and force limiting. The clinical trial of this study focused on measuring the contact force when pain occurred, as the biomechanical limits suggested by ISO/TS 15066 are defined from the onset of pain. The contact scenario aimed to mimic the real collision situation that could occur in the human-robot interaction. Two types of contact surfaces and two body locations were considered, and the load that led to the onset of mechanical pain was measured to establish the safety criteria. Thirty-seven subjects participated in the clinical trial and were instructed to indicate when they experienced pain during the trial. The measured contact forces were subjected to statistical analyses to derive the representative values as biomechanical limits. The distribution model was the log-logistics CDF, and percentiles were calculated based on the generated model.

The 75th percentile was extracted from the fitted log-logistic CDF and compared with the results of existing studies. The findings varied considerably owing to the different contact conditions used in each study. Given these differences, it is necessary to establish a method to integrate the results of relevant studies to obtain definitive biomechanical limits. Accumulating results from studies with the explicit purpose of obtaining force (or pressure) pain thresholds, such as this study, may improve current biomechanical limits calculated in part from literature surveys. Future studies can be aimed at exploring the force and pressure pain thresholds of all 29 body locations and investigating the correlation between the contact condition and force pain threshold. Moreover, the biophysical property of body location in contact or collision could be examined from measured data, such as contact force and displacement of tissue, from clinical trials. Biophysical properties can potentially be utilized for human-robot contact modeling to verify collision safety in advance (Shin et al., 2019). These improvements are expected to contribute to the development of technologies needed to establish safer human-robot interactions.

## Data Availability

The original contributions presented in the study are included in the article/Supplementary material, further inquiries can be directed to the corresponding author.
